# Supplementary material for: Transcriptomic Insights into the Response of the Olfactory Bulb to Selenium Treatment in a Mouse Model of Alzheimer’s Disease
Source: Int J Mol Sci. 2019 Jun 19;20(12):2998. doi: 10.3390/ijms20122998 (PMC6627505; doi:10.3390/ijms20122998)
Supplement: Supplementary file 1 [file ijms-20-02998-s001.pdf]

Supplementary material

Table S1. Total list of differential genes.

Gene	Pos	log FC	p-value	Description	Web
Adcyap1	chr17:93198904-93206539	1.490543456	3.83E-07	adenylate cyclase activating polypeptide 1	http://www.ncbi.nlm.nih.gov/gene/?term=Adcyap1
Lrg1	chr17:56119678-56121946	-3.089282474	8.50E-07	leucine-rich alpha-2-glycoprotein 1	http://www.ncbi.nlm.nih.gov/gene/?term=Lrg1
Anxa1	chr19:20373434-20390671	-2.516888201	1.23E-06	annexin A1	http://www.ncbi.nlm.nih.gov/gene/?term=Anxa1
Cd177	chr7:24743983-24760334	-3.576578987	1.23E-06	CD177 antigen	http://www.ncbi.nlm.nih.gov/gene/?term=Cd177
Vwf	chr6:125552936-125686679	-1.154755736	2.52E-06	Von Willebrand factor homolog	http://www.ncbi.nlm.nih.gov/gene/?term=Vwf
Areg	chr5:91139615-91148432	3.490844372	1.38E-05	amphiregulin	http://www.ncbi.nlm.nih.gov/gene/?term=Areg
Il1r1	chr1:40225014-40316192	-1.261880197	0.000109373	interleukin 1 receptor, type I	http://www.ncbi.nlm.nih.gov/gene/?term=Il1r1
Scgb3a1	chr11:49663595-49665118	-3.753347292	0.000532647	secretoglobin, family 3A, member 1	http://www.ncbi.nlm.nih.gov/gene/?term=Scgb3a1
fantom3_1110030K16	chr11:49663612-49665118	-3.91481365	0.000781867	fantom3_1110030K16	http://www.ncbi.nlm.nih.gov/nuccore/AK003988
Scg2	chr1:79434669-79440123	1.025908276	0.000781867	secretogranin II	http://www.ncbi.nlm.nih.gov/gene/?term=Scg2
Abca13	chr11:9191354-9684871	-4.747023343	0.001135973	ATP-binding cassette, sub-family A (ABC1), member 13	http://www.ncbi.nlm.nih.gov/gene/?term=Abca13
Th	chr7:142892776-142931128	1.311274939	0.001222258	tyrosine hydroxylase	http://www.ncbi.nlm.nih.gov/gene/?term=Th
Pglyrp1	chr7:18884690-18890438	-1.615045207	0.001312348	peptidoglycan recognition protein 1	http://www.ncbi.nlm.nih.gov/gene/?term=Pglyrp1
Bpifb1	chr2:154190818-154220343	-6.706571062	0.001563297	BPI fold containing family B, member 1	http://www.ncbi.nlm.nih.gov/gene/?term=Bpifb1
Gpx3	chr11:54902854-54910382	-1.902807558	0.001740861	glutathione peroxidase 3	http://www.ncbi.nlm.nih.gov/gene/?term=Gpx3
Dlk1	chr12:109452823-109463336	-2.512195642	0.001740861	delta-like 1 homolog (Drosophila)	http://www.ncbi.nlm.nih.gov/gene/?term=Dlk1
Gabrp	chr11:33550781-33578982	-6.280066099	0.001740861	gamma-aminobutyric acid (GABA) A receptor, pi	http://www.ncbi.nlm.nih.gov/gene/?term=Gabrp
Fabp7	chr10:57784923-57788450	1.294464203	0.001890748	fatty acid binding protein 7, brain	http://www.ncbi.nlm.nih.gov/gene/?term=Fabp7
Serpina3n	chr12:104406708-104414329	-1.202384973	0.001890748	serine (or cysteine) peptidase inhibitor, clade A, member 3N	http://www.ncbi.nlm.nih.gov/gene/?term=Serpina3n
S100a11	chr3:93520406-93526288	-1.284722188	0.001942156	S100 calcium binding protein A11 (calgizzarin)	http://www.ncbi.nlm.nih.gov/gene/?term=S100a11
Muc5b	chr7:141838946-141873085	-6.351221378	0.001942156	mucin 5, subtype B, tracheobronchial	http://www.ncbi.nlm.nih.gov/gene/?term=Muc5b
Lypd2	chr15:74732246-74734795	-5.614288167	0.001942156	Ly6/Plaur domain containing 2	http://www.ncbi.nlm.nih.gov/gene/?term=Lypd2
Pon1	chr6:5168090-5193987	-7.076559306	0.00222768	paraoxonase 1	http://www.ncbi.nlm.nih.gov/gene/?term=Pon1
Il18r1	chr1:40465552-40500854	-2.448319543	0.003641394	interleukin 18 receptor 1	http://www.ncbi.nlm.nih.gov/gene/?term=Il18r1
Ccdc3	chr2:5137776-5230871	-1.415421734	0.0037825	coiled-coil domain containing 3	http://www.ncbi.nlm.nih.gov/gene/?term=Ccdc3
Krt5	chr15:101707070-101712891	-7.573359446	0.0037825	keratin 5	http://www.ncbi.nlm.nih.gov/gene/?term=Krt5

Slc39a4	chr15:76612383-76617379	-3.42185642	0.004608117	solute carrier family 39 (zinc transporter), member 4	http://www.ncbi.nlm.nih.gov/gene/?term=Slc39a4
fantom3_6330419P03	chr19:5842304-5843844	-1.175883043	0.005335604	fantom3_6330419P03	http://www.ncbi.nlm.nih.gov/nuccore/AK018202
Ifitm1	chr7:140967429-140969828	-2.061519347	0.006176521	interferon induced transmembrane protein 1	http://www.ncbi.nlm.nih.gov/gene/?term=Ifitm1
Galnt15	chr14:32028651-32060375	-1.849241631	0.006176521	UDP-N-acetyl-alpha-D-galactosamine:polypeptide N-acetylgalactosaminyltransferase 15	http://www.ncbi.nlm.nih.gov/gene/?term=Galnt15
Bpifa1	chr2:154142880-154149217	-7.422792623	0.006176521	BPI fold containing family A, member 1	http://www.ncbi.nlm.nih.gov/gene/?term=Bpifa1
Pip5k1b	chr19:24294794-24555827	1.034832706	0.007004973	phosphatidylinositol-4-phosphate 5-kinase, type 1 beta	http://www.ncbi.nlm.nih.gov/gene/?term=Pip5k1b
Trhr2	chr8:122356967-122360746	1.698942015	0.00807457	thyrotropin releasing hormone receptor 2	http://www.ncbi.nlm.nih.gov/gene/?term=Trhr2
Etv5	chr16:22381313-22439723	1.026873634	0.00807457	ets variant gene 5	http://www.ncbi.nlm.nih.gov/gene/?term=Etv5
Tmem30b	chr12:73543114-73546395	-5.000356112	0.008164102	transmembrane protein 30B	http://www.ncbi.nlm.nih.gov/gene/?term=Tmem30b
Agxt2l1	chr3:130617448-130635750	-1.062205129	0.009042465	alanine-glyoxylate aminotransferase 2-like 1	http://www.ncbi.nlm.nih.gov/gene/?term=Agxt2l1
Ehf	chr2:103263431-103303275	-3.164335883	0.010962526	ets homologous factor	http://www.ncbi.nlm.nih.gov/gene/?term=Ehf
Cyp2f2	chr7:27119955-27133660	-5.55530174	0.010962526	cytochrome P450, family 2, subfamily f, polypeptide 2	http://www.ncbi.nlm.nih.gov/gene/?term=Cyp2f2
fantom3_G530010F21	chr17:17887851-17890807	-6.558674324	0.011273945	fantom3_G530010F21	http://www.ncbi.nlm.nih.gov/nuccore/AK149690
Lbp	chr2:158306493-158332852	-2.375494221	0.011728699	lipopolysaccharide binding protein	http://www.ncbi.nlm.nih.gov/gene/?term=Lbp
fantom3_1110005E01	chr16:22381310-22382629	1.005091791	0.012986845	fantom3_1110005E01	http://www.ncbi.nlm.nih.gov/nuccore/AK003461
Bpifb9b	chr2:154307244-154320644	-6.744634671	0.012986845	BPI fold containing family B, member 9B	http://www.ncbi.nlm.nih.gov/gene/?term=Bpifb9b
Bpifb9a	chr2:154256735-154271764	-6.154853768	0.013574667	BPI fold containing family B, member 9A	http://www.ncbi.nlm.nih.gov/gene/?term=Bpifb9a
Wfdc2	chr2:164562564-164568510	-4.635632824	0.014169418	WAP four-disulfide core domain 2	http://www.ncbi.nlm.nih.gov/gene/?term=Wfdc2
Clca2	chr3:145070260-145099500	-4.755974729	0.014293683	chloride channel calcium activated 2	http://www.ncbi.nlm.nih.gov/gene/?term=Clca2
Fcamr	chr1:130785205-130825795	-7.194983401	0.014293683	Fc receptor, IgA, IgM, high affinity	http://www.ncbi.nlm.nih.gov/gene/?term=Fcamr
Tff2	chr17:31141062-31144282	-6.185587816	0.014570157	trefoil factor 2 (spasmolytic protein 1)	http://www.ncbi.nlm.nih.gov/gene/?term=Tff2
Col28a1	chr6:7997111-8192617	1.335370441	0.014570157	collagen, type XXVIII, alpha 1	http://www.ncbi.nlm.nih.gov/gene/?term=Col28a1
Wfdc18	chr11:83709004-83711360	-5.23061309	0.014570157	WAP four-disulfide core domain 18	http://www.ncbi.nlm.nih.gov/gene/?term=Wfdc18
fantom3_D930027K06	chr3:79641611-79643735	1.775046945	0.014865539	fantom3_D930027K06	http://www.ncbi.nlm.nih.gov/nuccore/AK086423
fantom3_D930001D16	chr3:79641611-79643735	1.775047128	0.014865539	fantom3_D930001D16	http://www.ncbi.nlm.nih.gov/nuccore/AK142756
Rtp1	chr16:23428601-23433960	-3.239085351	0.014865539	receptor transporter protein 1	http://www.ncbi.nlm.nih.gov/gene/?term=Rtp1
Aox3l1	chr1:58278326-58379264	-8.298167467	0.014865539	aldehyde oxidase 3-like 1	http://www.ncbi.nlm.nih.gov/gene/?term=Aox3l1
Krt18	chr15:102022706-102032026	-5.131742908	0.016056199	keratin 18	http://www.ncbi.nlm.nih.gov/gene/?term=Krt18
Gm10635	chr9:79444037-79519302	2.918646276	0.016349005	predicted gene 10635	http://www.ncbi.nlm.nih.gov/gene/?term=Gm10635

Tacstd2	chr6:67534059-67535822	-3.133417388	0.016349005	tumor-associated calcium signal transducer 2	http://www.ncbi.nlm.nih.gov/gene/?term=Tacstd2
Cyp4b1	chr4:115624728-115647705	-5.030589083	0.016349005	cytochrome P450, family 4, subfamily b, polypeptide 1	http://www.ncbi.nlm.nih.gov/gene/?term=Cyp4b1
Nppa	chr4:148000746-148002067	1.202726969	0.016349005	natriuretic peptide type A	http://www.ncbi.nlm.nih.gov/gene/?term=Nppa
Foxa1	chr12:57540628-57548029	-6.173003203	0.016349005	forkhead box A1	http://www.ncbi.nlm.nih.gov/gene/?term=Foxa1
Spata18	chr5:73651380-73679484	-4.529546493	0.016458847	spermatogenesis associated 18	http://www.ncbi.nlm.nih.gov/gene/?term=Spata18
fantom3_2310043N10	chr19:5842309-5844407	-1.063413228	0.016596015	fantom3_2310043N10	please add 2310043N10 into http://www.ncbi.nlm.nih.gov/nuccore/
Sult1c1	chr17:53961615-53990631	-5.687350223	0.016789696	sulfotransferase family, cytosolic, 1C, member 1	http://www.ncbi.nlm.nih.gov/gene/?term=Sult1c1
Pigr	chr1:130826684-130852249	-5.615041921	0.017122408	polymeric immunoglobulin receptor	http://www.ncbi.nlm.nih.gov/gene/?term=Pigr
Aqp3	chr4:41092724-41098183	-5.877145191	0.018384376	aquaporin 3	http://www.ncbi.nlm.nih.gov/gene/?term=Aqp3
Olfm4	chr14:80000154-80023136	-1.905503324	0.01878593	olfactomedin 4	http://www.ncbi.nlm.nih.gov/gene/?term=Olfm4
Ugt1a6b	chr1:88103257-88218998	-1.948806921	0.01878593	UDP glucuronosyltransferase 1 family, polypeptide A6B	http://www.ncbi.nlm.nih.gov/gene/?term=Ugt1a6b
Ugt1a2	chr1:88200611-88220002	-1.927197922	0.01878593	UDP glucuronosyltransferase 1 family, polypeptide A2	http://www.ncbi.nlm.nih.gov/gene/?term=Ugt1a2
Sntn	chr14:13670876-13683148	-8.135777705	0.019391191	sentan, cilia apical structure protein	http://www.ncbi.nlm.nih.gov/gene/?term=Sntn
Slc5a9	chr4:111875374-111902796	-6.777390073	0.019391191	solute carrier family 5 (sodium/glucose cotransporter), member 9	http://www.ncbi.nlm.nih.gov/gene/?term=Slc5a9
Ugt1a7c	chr1:88095001-88220002	-2.00182524	0.02018632	UDP glucuronosyltransferase 1 family, polypeptide A7C	http://www.ncbi.nlm.nih.gov/gene/?term=Ugt1a7c
Ugt1a5	chr1:88166012-88220002	-1.927951688	0.02018632	UDP glucuronosyltransferase 1 family, polypeptide A5	http://www.ncbi.nlm.nih.gov/gene/?term=Ugt1a5
fantom3_F430109O12	chr7:27124481-27130674	-5.96437461	0.020487445	fantom3_F430109O12	http://www.ncbi.nlm.nih.gov/nuccore/AK143796
Wdr95	chr5:149484260-149611894	-4.212301707	0.020570366	WD40 repeat domain 95	http://www.ncbi.nlm.nih.gov/gene/?term=Wdr95
Ugt1a6a	chr1:88134809-88220002	-1.82120873	0.020570366	UDP glucuronosyltransferase 1 family, polypeptide A6A	http://www.ncbi.nlm.nih.gov/gene/?term=Ugt1a6a
Hsd17b6	chr10:127990936-128007508	-7.186664718	0.020795118	hydroxysteroid (17-beta) dehydrogenase 6	http://www.ncbi.nlm.nih.gov/gene/?term=Hsd17b6
Spag16	chr1:69826872-70725132	-3.030342099	0.020931389	sperm associated antigen 16	http://www.ncbi.nlm.nih.gov/gene/?term=Spag16
Ugt1a9	chr1:88070779-88220002	-1.919850462	0.021093464	UDP glucuronosyltransferase 1 family, polypeptide A9	http://www.ncbi.nlm.nih.gov/gene/?term=Ugt1a9
Ugt1a10	chr1:88055411-88220002	-1.919850597	0.021093464	UDP glycosyltransferase 1 family, polypeptide A10	http://www.ncbi.nlm.nih.gov/gene/?term=Ugt1a10
Ugt1a1	chr1:88211959-88220002	-1.928574399	0.021093464	UDP glucuronosyltransferase 1 family, polypeptide A1	http://www.ncbi.nlm.nih.gov/gene/?term=Ugt1a1
Tspan1	chr4:116161881-116167598	-4.101104137	0.021348373	tetraspanin 1	http://www.ncbi.nlm.nih.gov/gene/?term=Tspan1
Ak7	chr12:105705668-105782447	-3.999810224	0.021614257	adenylate kinase 7	http://www.ncbi.nlm.nih.gov/gene/?term=Ak7

Serpinb11	chr1:107362314-107380475	-6.049173769	0.021862355	serine (or cysteine) peptidase inhibitor, clade B (ovalbumin), member 11	http://www.ncbi.nlm.nih.gov/gene/?term=Serpinb11
fantom3_9030612O11	chr4:115624728-115640428	-4.802594285	0.02269057	fantom3_9030612O11	http://www.ncbi.nlm.nih.gov/nuccore/AK078882
Fetub	chr16:22918382-22939768	-4.745417605	0.022734237	fetuin beta	http://www.ncbi.nlm.nih.gov/gene/?term=Fetub
Ildr1	chr16:36693978-36726804	-2.842165139	0.022734237	immunoglobulin-like domain containing receptor 1	http://www.ncbi.nlm.nih.gov/gene/?term=Ildr1
Sec14l3	chr11:4064853-4078990	-6.369711258	0.022734237	SEC14-like 3 (S. cerevisiae)	http://www.ncbi.nlm.nih.gov/gene/?term=Sec14l3
Six1	chr12:73041827-73046712	-3.124944547	0.023663259	sine oculis-related homeobox 1	http://www.ncbi.nlm.nih.gov/gene/?term=Six1
Cbr2	chr11:120729485-120732026	-4.34947418	0.02500231	carbonyl reductase 2	http://www.ncbi.nlm.nih.gov/gene/?term=Cbr2
Ces1d	chr8:93166072-93197804	-5.407403418	0.02569644	carboxylesterase 1D	http://www.ncbi.nlm.nih.gov/gene/?term=Ces1d
Chi3l4	chr3:106201491-106219479	-10.1448513	0.02665744	chitinase 3-like 4	http://www.ncbi.nlm.nih.gov/gene/?term=Chi3l4
Muc20	chr16:32777419-32797435	-5.953878293	0.028464822	mucin 20	http://www.ncbi.nlm.nih.gov/gene/?term=Muc20
Slc44a4	chr17:34914239-34930436	-5.884397221	0.029011624	solute carrier family 44, member 4	http://www.ncbi.nlm.nih.gov/gene/?term=Slc44a4
Cldn7	chr11:69964779-69967886	-3.53851712	0.029011624	claudin 7	http://www.ncbi.nlm.nih.gov/gene/?term=Cldn7
St14	chr9:31088590-31131894	-2.472908405	0.029011624	suppression of tumorigenicity 14 (colon carcinoma)	http://www.ncbi.nlm.nih.gov/gene/?term=St14
Sftpd	chr14:41172212-41185198	-5.329604608	0.029011624	surfactant associated protein D	http://www.ncbi.nlm.nih.gov/gene/?term=Sftpd
fantom3_2310042I22	chr1:162874322-162876094	-1.794829453	0.029011624	fantom3_2310042I22	http://www.ncbi.nlm.nih.gov/nuccore/AK009753
fantom3_A730072G01	chr7:18884693-18885700	-1.421877078	0.029257185	fantom3_A730072G01	http://www.ncbi.nlm.nih.gov/nuccore/AK043227
1810011O10Rik	chr8:24437616-24438946	-1.538080818	0.029257185	RIKEN cDNA 1810011O10 gene	http://www.ncbi.nlm.nih.gov/gene/?term=1810011O10Rik
Inmt	chr6:55170627-55174990	-3.064935829	0.029710235	indolethylamine N-methyltransferase	http://www.ncbi.nlm.nih.gov/gene/?term=Inmt
Svopl	chr6:37983739-38047025	-3.502962214	0.029710235	SV2 related protein homolog (rat)-like	http://www.ncbi.nlm.nih.gov/gene/?term=Svopl
Dnahc6	chr6:73017607-73221631	-2.989645327	0.03391448	Dnahc6	http://www.ncbi.nlm.nih.gov/gene/?term=Dnahc6
Pitx1	chr13:55825044-55836195	-4.561279694	0.034272156	paired-like homeodomain transcription factor 1	http://www.ncbi.nlm.nih.gov/gene/?term=Pitx1
Krt14	chr11:100203162-100207510	-5.114521918	0.035422749	keratin 14	http://www.ncbi.nlm.nih.gov/gene/?term=Krt14
Cd44	chr2:102811141-102901673	-1.470529123	0.035422749	CD44 antigen	http://www.ncbi.nlm.nih.gov/gene/?term=Cd44
AU040972	chr11:79481723-79484031	-3.939000536	0.035422749	expressed sequence AU040972	http://www.ncbi.nlm.nih.gov/gene/?term=AU040972
Gsta3	chr1:21240589-21265559	-2.570550575	0.035489626	glutathione S-transferase, alpha 3	http://www.ncbi.nlm.nih.gov/gene/?term=Gsta3
Nt5c1a	chr4:123201201-123225182	1.368641533	0.035690125	5'-nucleotidase, cytosolic IA	http://www.ncbi.nlm.nih.gov/gene/?term=Nt5c1a
fantom3_A130022C14	chr11:54445376-54447787	-6.038178594	0.035690125	fantom3_A130022C14	http://www.ncbi.nlm.nih.gov/nuccore/AK037500
C1s	chr6:124530344-124542359	-1.967007127	0.035739236	complement component 1, s subcomponent	http://www.ncbi.nlm.nih.gov/gene/?term=C1s
Gal	chr19:3409917-3414457	1.484695982	0.037214701	galanin	http://www.ncbi.nlm.nih.gov/gene/?term=Gal
Tmprss2	chr16:97564682-97659548	-4.190526868	0.037929983	transmembrane protease, serine 2	http://www.ncbi.nlm.nih.gov/gene/?term=Tmprss2
Fam46b	chr4:133480133-133487940	-1.825624014	0.03819662	family with sequence similarity 46, member B	http://www.ncbi.nlm.nih.gov/gene/?term=Fam46b
fantom3_A430024L20	chr13:93358589-93360525	1.119221006	0.038522591	fantom3_A430024L20	http://www.ncbi.nlm.nih.gov/nuccore/AK039878
Msln	chr17:25748613-25754446	-2.595345926	0.038918951	mesothelin	http://www.ncbi.nlm.nih.gov/gene/?term=Msln

Cldn8	chr16:88560826-88563183	-5.040473472	0.038955074	claudin 8	http://www.ncbi.nlm.nih.gov/gene/?term=Cldn8
fantom3_D130051D11	chr15:85665282-85667805	-1.445259043	0.038955074	fantom3_D130051D11	http://www.ncbi.nlm.nih.gov/nuccore/AK083890
Obp2a	chr2:25697538-25703332	-9.333874825	0.039651208	odorant binding protein 2A	http://www.ncbi.nlm.nih.gov/gene/?term=Obp2a
Scnn1a	chr6:125321340-125344943	-2.277931389	0.039651208	sodium channel, nonvoltage-gated 1 alpha	http://www.ncbi.nlm.nih.gov/gene/?term=Scnn1a
Olfr70	chr4:43695000-43700807	-6.718107316	0.039651208	olfactory receptor 70	http://www.ncbi.nlm.nih.gov/gene/?term=Olfr70
Elf5	chr2:103411693-103450989	-4.481014142	0.040273235	E74-like factor 5	http://www.ncbi.nlm.nih.gov/gene/?term=Elf5
Steap4	chr5:7960472-7982213	-2.384918372	0.040273235	STEAP family member 4	http://www.ncbi.nlm.nih.gov/gene/?term=Steap4
fantom3_G730026I11	chr19:4062439-4065879	-1.198983549	0.040323921	fantom3_G730026I11	http://www.ncbi.nlm.nih.gov/nuccore/AK144717
Reg3g	chr6:78466268-78468874	-4.434363489	0.040936943	regenerating islet-derived 3 gamma	http://www.ncbi.nlm.nih.gov/gene/?term=Reg3g
Epcam	chr17:87635979-87651127	-4.081830154	0.041557445	epithelial cell adhesion molecule	http://www.ncbi.nlm.nih.gov/gene/?term=Epcam
Rxfp1	chr3:79643990-79737949	1.733658837	0.041599893	relaxin/insulin-like family peptide receptor 1	http://www.ncbi.nlm.nih.gov/gene/?term=Rxfp1
Ccdc153	chr9:44240655-44247306	-4.757925794	0.041599893	coiled-coil domain containing 153	http://www.ncbi.nlm.nih.gov/gene/?term=Ccdc153
Agr2	chr12:35992925-36004081	-5.821893422	0.041677237	anterior gradient 2 (Xenopus laevis)	http://www.ncbi.nlm.nih.gov/gene/?term=Agr2
Clic6	chr16:92498147-92541241	-2.536858518	0.042956145	chloride intracellular channel 6	http://www.ncbi.nlm.nih.gov/gene/?term=Clic6
fantom3_C330006P03	chr13:93358401-93360457	1.12174187	0.042956145	fantom3_C330006P03	http://www.ncbi.nlm.nih.gov/nuccore/AK049142
Fam216b	chr14:78081024-78089007	-4.096717006	0.042956145	family with sequence similarity 216, member B	http://www.ncbi.nlm.nih.gov/gene/?term=Fam216b
St6galnac1	chr11:116765025-116775507	-1.94227853	0.042956145	ST6 (alpha-N-acetyl-neuraminyl-2,3-beta-galactosyl-1,3)-N-acetylgalactosaminide alpha-2,6-sialyltransferase 1	http://www.ncbi.nlm.nih.gov/gene/?term=St6galnac1
BC051019	chr7:109712181-109723937	-3.400125439	0.043799327	cDNA sequence BC051019	http://www.ncbi.nlm.nih.gov/gene/?term=BC051019
Tnfsf15	chr4:63724603-63745113	2.767933558	0.044088036	tumor necrosis factor (ligand) superfamily, member 15	http://www.ncbi.nlm.nih.gov/gene/?term=Tnfsf15
fantom3_1110069O07	chr11:4078145-4078990	-5.653447014	0.044214579	fantom3_1110069O07	http://www.ncbi.nlm.nih.gov/nuccore/AK004424
fantom3_1810073O08	chr17:83917937-83921606	-1.990194924	0.04595683	fantom3_1810073O08	http://www.ncbi.nlm.nih.gov/nuccore/AK007978
Dmbt1	chr7:131032057-131121628	-6.628279278	0.046451504	deleted in malignant brain tumors 1	http://www.ncbi.nlm.nih.gov/gene/?term=Dmbt1
Mapk13	chr17:28769317-28778704	-2.904492457	0.046451504	mitogen-activated protein kinase 13	http://www.ncbi.nlm.nih.gov/gene/?term=Mapk13
S100a5	chr3:90606480-90611784	1.622485359	0.046451504	S100 calcium binding protein A5	http://www.ncbi.nlm.nih.gov/gene/?term=S100a5
Mup4	chr4:59956806-59960665	-9.001545866	0.04765892	major urinary protein 4	http://www.ncbi.nlm.nih.gov/gene/?term=Mup4
Obp2b	chr2:25714213-25740115	-6.148074213	0.047848075	odorant binding protein 2B	http://www.ncbi.nlm.nih.gov/gene/?term=Obp2b
fantom3_C130018M10	chr10:23102963-23104326	-1.761539052	0.049090936	fantom3_C130018M10	http://www.ncbi.nlm.nih.gov/nuccore/AK081454
1190005I06Rik	chr8:120608602-120634382	-1.040128985	0.049325167	RIKEN cDNA 1190005I06 gene	http://www.ncbi.nlm.nih.gov/gene/?term=1190005I06Rik
Cdhr3	chr12:33033796-33093040	-3.104758397	0.049745564	cadherin-related family member 3	http://www.ncbi.nlm.nih.gov/gene/?term=Cdhr3
